# Engaging the normative question in the H5N1 avian influenza mutation experiments

**DOI:** 10.1186/1747-5341-8-12

**Published:** 2013-09-05

**Authors:** Norman K Swazo

**Affiliations:** 1 College of Science and General Studies, Alfaisal University, PO Box 50927, Riyadh 11533, Saudi Arabia

**Keywords:** Influenza, Fouchier, Kawaoka, NSABB, H5N1, Ethics

## Abstract

**Introduction:**

In recent time there has been ample discussion concerning censorship of research conducted in two labs involved in avian influenza virus research. Much of the debate has centered on the question whether the methods and results should reach to open disclosure given the “dual use” nature of this research which can be used for nefarious purposes.

**Methods:**

This paper reviews the discussion to date but centers on epistemological issues associated with initial justification of this research and what this entails for continuation of this research despite US governmental biosecurity concerns. The question here is whether there was reasonable moral warrant for genetic alteration of the H5N1 influenza virus.

**Conclusion:**

The paper concludes with philosophical (ethical) justification for continuation of this research.

## Introduction

### The ethical question at issue

The year 2011 ended with significant concern about prospective publication of troubling results of research on a strain of H5N1 avian influenza virus, such that reputable scientists characterized the scene of disputation as one of “life sciences at a crossroads.” Even *The New York Times* published an editorial warning of impending catastrophic harm, “an engineered doomsday” [1,2]. Both *Nature* and *Science* journals were initially faced with an extraordinary editorial decision, following a request from the U.S. National Science Advisory Board for Biosecurity (NSABB) to restrict disclosure of the details of two experiments from two research teams (Fouchier, Kawaoka, as described below), based on NSABB’s review of the initial papers submitted. The U.S. Department of Health and Human Services concurred with the recommendation. The request was unprecedented in the annals of scientific research. NSABB was concerned specifically with one possibility: “Could this knowledge, in the hands of malevolent individuals, organizations, or governments, allow construction of a genetically altered influenza virus capable of causing a pandemic with mortality exceeding that of the ‘Spanish flu’ epidemic of 1918?” [3].

The norm of open publication of research, of course, allows access to the experimental methods and prospective replication of research results, consistent with peer review and international advancement of the particular research agenda. The initial NSABB request would restrict such open publication. According to NSABB’s initial risk assessment in this case, “the potential risk of public harm [is] of unusually high magnitude” [3]. The question being asked initially was whether the journals should defer to the NSABB and effectively redact submitted papers to satisfy the biosecurity concerns. Perhaps equally as important, the controversy over publication suffered from relevant legal oversight mechanisms, even though “the international scale of such research brings international law into the picture” [4].

The question about full or redacted publication is, of course, important to ask from the perspective of national (and even “global”) biosecurity policy. This policy already presupposes the legitimacy of dual use research, hence the NSABB institutional oversight in this case. Further, such policy also presupposes adequate biosafety measures in place in laboratory settings, to prevent or mitigate biologic threats to the national public health and/or national security—not to mention threats to global health and international security involving microbial agents that could be weaponized or released inadvertently into the environment. But, in present case there is at least one prior, more fundamental, question to be asked. This is not an empirical question, in the sense that one may be concerned with the success or failure of the particular research methods in use or the outcomes of this research for translational medicine, e.g., production of a vaccine or antivirals. Neither is it a question about restrictions on publication such as both *Nature* and *Science* editors have faced. Rather, the more fundamental question is normative, and it is grounded in the conventions of biomedical research ethics. The key question then is:

Is there moral warrant in place reasonably to permit genetic alteration of the H5N1 strain of avian influenza for the purposes presented in the research protocol?

This is the question engaged in the following discussion. This paper thus contributes to the NSABB call for a broad and international engagement of the issues raised by the Fouchier and Kawaoka experiments.

## Results and discussion

### Summarizing the debate

Two studies were involved, one from a research team led by virologist Ron Fouchier at the Erasmus Medical Center in Rotterdam, Netherlands, involving genetic engineering of H5N1; the other led by virologist Yoshihiro Kawaoka of the University of Wisconsin-Madison School of Veterinary Medicine (Institute for Influenza Viral Research) and the University of Tokyo (Institute of Medical Science), this research involving H5N1-H1N1 reassortment. Both studies are sub-contracts for a National Institute of Health/National Institute for Allergy and Infectious Diseases (NIH-NIAID) grant award that established an NIAID Center of Excellence for Influenza Research and Surveillance (CEIRS), specifically a Center for Research on Influenza Pathogenesis (CRIP) at Mt. Sinai School of Medicine (Research Area: Pathogenesis and Host Response Research; Principal Investigator: Adolfo Garcia-Sastre).

These two experiments succeeded in genetically altering H5N1, with the effect in the Fouchier experiment of aerosol (airborne) transmission of the mutated virus in ferrets^a^. It is claimed that ferrets provide an animal model most similar to humans for study of H5N1 disease transmission. Airborne transmission is not now a natural occurrence for H5N1 incidence in humans. Hence, the chief public health and “biosecurity” concern consequent to this research is inadvertent release of a “man-made virus” into the environment, and thus a prospective pandemic having high human mortality.

Berns *et. al.* report that the Fouchier experiment exhibited transmission “with maintenance of high pathogenicity” [3]. However, Fouchier emphasized his experiment is not reasonably to be construed as a quantitative model for transmission—i.e., while the experiment has indeed shown aerosol transmission, the *efficiency* of transmission cannot be deduced from the experiment’s results. (Personal notes, web-link access to AMS presentation; see reference [5]). This point is important further in clarifying misperceptions reported in the public press about the degree of pathogenicity of the H5N1 mutation. In a comparison of virus titer of pH1N1 [pandemic H1N1] and the mutated form of H5N1 over 1–5 days of infection, Fouchier reports, the pH1N1 shows massive replication after inoculation exposure as early as Day 1 in the recipient ferret, and generally all ferrets start to shed virus in high amounts by Day 2 (Personal notes, web-link access to AMS presentation; see reference [5]). Fouchier argued that, in contrast to the pH1N1 data: H5N1 does not transmit in 100% of the cases; virus titers are much lower; many of the ferrets do not start shedding virus until Day 3 or 5; and therefore, the H5N1 mutation does not spread like pH1N1 or seasonal influenza. Moreover, the pathogenicity of the mutated H5N1 varies: When the dose of H5N1-Wt (wild type) virus is at 10^6^ and the inoculation route is *intranasal*, 1 out of 8 ferrets was dead or moribund by 6 days post-exposure; when H5N1-Mut transmission is by *aerosol*, 0 out of 7 ferrets are dead or moribund; but, when the H5N1-Mut inoculation route is *intratracheal*, 6 out of 6 ferrets were dead or moribund by day 3 post-exposure. Fouchier stressed further that when ferrets have been pre-exposed to seasonal flu there is no lethality at 10^6^ dose—that is, the pre-exposure provides cross-protective immunity.

Some NSABB infectious disease specialists are not satisfied Fouchier’s remarks alter the risk-benefit assessment, given both the changed host range and changed mode of transmission of H5N1-Mut [6]. H5N1 is of particular concern because a strain of H5N1 avian-origin virus adapted to humans and caused the 1918–1919 pandemic with an estimated 40–50 million deaths worldwide [7]. The adaptations of such viruses occur “gradually by point mutation (antigenic drift) or drastically by genetic reassortment (antigenic shift)” [8]. Further, avian influenza is particularly problematic for the agricultural sector of the economy. In Pennsylvania USA, e.g., eradication of an avirulent H5N2 influenza virus in chickens required “destruction of over 17 million birds at a cost of over 61 million dollars” [8,9]. In the case of the Hong Kong outbreak of H5N1, the costs are estimated in the US$100s millions, with the global loss from avian influenza estimated in the billions since 2003 [10,11].

Epidemiological data for H5N1 are in dispute, even as some argue the dispute centers on “the wrong questions” [12]. Some data show H5N1 has a “high virulence in both avian and mammalian species,” having been transmitted “from birds in live poultry markets in Hong Kong to humans,” with corresponding high mortality rate in those humans infected when it surfaced in 1997 [8,13]. The virus has exhibited sporadic occurrence in humans in the intervening period, but with an estimated 60% case fatality rate, although infection in humans is rare given the current mode of transmission [7,14].

Fouchier *et. al.,* report that, as of January 2012, “HPAI H5N1 virus caused 577 laboratory-confirmed human cases of infection, of which 340 were fatal,” although “sustained human-to-human transmission has not been reported” [15]. Peter Palese argues that the case fatality ratio estimate is “too high, because only severe [hospital] cases are being counted;” and “People who are asymptomatically infected are not being counted” [16]. In short, argue Wang *et. al.,* “The prevalence of avian H5N1 influenza A infections in humans has not been definitively determined,” although the claim here is that the likely infection rate in exposed populations is 1-2%, H5N1 thereby having a much lower case-fatality ratio than estimated by WHO [17,18]. This latter claim, however, is itself countered by Osterholm and Kelley, given that “case-finding strategies may influence the overall case-fatality rate reported by the WHO;” but they argue that the “available seroepidemiological data for H5N1 infection support the current WHO-reported case-fatality rates of 30% to 80%” [19].

Despite the above observations, NSABB provided a *utilitarian* argument with a view to maximizing benefits and minimizing risk: “Because the NSABB found that there was significant potential for harm in fully publishing these results and that the harm exceeded the benefits of publication, we therefore recommended that the work not be fully communicated in an open forum” [3]. NSABB also gave priority appeal to *non-maleficence* over beneficence in evaluating researcher responsibilities, even as they pointed to the need for *prudence* in this case. While Paul Keim, acting chair of NSABB, acknowledged bioterrorist uses of this modified virus are of “low probability,” he is nonetheless concerned that a terrorist “could introduce a new evolutionary seed into the environment that seems not to exist in nature. This might not cause a pandemic instantly, but it could start the virus on a new path for pandemic evolution” [20-23].

Keim compared H5N1 with H1N1, which caused a low-virulence influenza pandemic in 2009, noting that the latter “was impossible to contain,” and asserted “the same would be true for an H5N1 influenza pandemic,” this one of more concern because of its high virulence. Keim’s concerns are not without scientific grounding, given what has been learned from the 1918 influenza strain genetic analyses. Taubenberger *et. al.* take note of “amino acid changes identified in the 1918 analysis” that are “also seen in HPAI strains of H5N1…viruses;” and they opine that, (1) “these changes may facilitate virus replication in human cells and increase pathogenicity;” (2) “the high pathogenicity of the 1918 virus was related to its emergence as a human-adapted avian influenza virus;” and (3) “the 1918 virus was most likely not a human/avian reassortment virus, but rather an avian-like virus that adapted to humans *in toto*” [24].

The editor-in-chief of *Science* issued a statement summarizing the initial NSABB request [25]. In tension here is (a) “the need to know” normally asserted and expected by scientists engaged in responsible influenza research, for the purpose of disease control and prevention; and (b) the prospect of dual-use research of concern (DURC) having deleterious “biosecurity” consequences in the event someone decides to weaponize the H5N1 virus consequent to knowledge of the methods of genetic alteration. Consequent to the NSABB request, Fouchier and Kawaoka implemented a “voluntary pause of 60 days” of their research activities [26,27]. One additional consequence of this research publication impasse is that the U.S. National Security Council has sought “greater federal control” over such studies [28]. On 29 March 2012, the US government issued a new policy for oversight of life sciences “dual use research of concern” (DURC) [29]. The U.S. Office of Biotechnology defines “dual use” research as “biological research with legitimate scientific purpose that may be misused to pose a biologic threat to public health and/or national security” [30]. After much international discussion and a second round of NSABB review of revised manuscripts, at a meeting held on 29–30 March 2012, NSABB voted unanimously to recommend full publication of the revised Kawaoka paper and voted 12–6 to recommend full publication of the revised Fouchier paper [31]. These recommendations were forwarded to the Department of Health and Human Services for final determination. Kawaoka *et. al*. published their unredacted version of the paper in early May 2012 [32]. Fouchier’s team published in June 2012 [33].

As bioethicists know, the moral question at issue here can be engaged according to various theoretical frameworks of moral analysis that unavoidably combine both the empirical data and moral principles and methods that guide normative assessment. The fact is—or so it seems from information available to the public at large—that the question of moral warrant posed above had not been asked in these particular research projects prior to the fact, though it was raised once the issue of publication of results came to the fore [34,35]. This is surely problematic from the perspective of research ethics, hence the need for the analysis engaged here. Moreover, the entire debate about open disclosure of the research methods associated with the two experiments, even if disclosure were to be restricted to those authorized on criteria of “need to know,” presupposes opportunity for replication of the research and the usual “falsification/verification” of the Fouchier and Kawaoka results, in which case the moral question above has even further urgency: *If the warrant for the original experiments is lacking, then* a fortiori *so is the warrant for consequent experiments very likely to be wanting.* Hans-Jörg Ehni wrote in 2008, “In the beginning of the 21st century, the technology which raises the most concern is the genetic construction and reconstruction of pathogenic organisms and viruses by biotechnological means” [36]. H5N1 research now raises this concern fully, whatever the risk/benefit calculation issued at any given time.

### Applicable biosafety protocol

The Center for Disease Control and Prevention has established technical guidelines for such research [37]. The operative term is *containment*, involving “safe methods, facilities and equipment for managing infectious materials in the laboratory environment where they are being handled or maintained” [37]. Such methods, facilities, and equipment are to assure appropriate *risk assessment*, ‘risk’ here understood to mean “exposure” to “potentially hazardous agents.” Risk is mitigated—reduced or eliminated—through the controls on exposure. These controls are specified according to various *biosafety levels* (Class 1, 2, and 3). Class 3 biological safety “provides the highest attainable level of protection to personnel and the environment” [37]. Biosafety levels also incorporate various specifications of “primary barriers” and “personal protective equipment” (e.g., containers, safety cabinets, gas-tight safety cabinets, full-body air-supplied positive-pressure personnel suits, clothing change protocols) and “secondary barriers” obtained through “facility design and construction” (e.g., controlled access, decontamination facility, specialized ventilation, airlocks and air treatment). There are also recommended biosafety levels set for particular microbial agents being handled, consistent with known data about “virulence, pathogenicity, antibiotic resistance patterns, vaccine and treatment availability.”

We are informed that the two research projects at issue here were conducted under *Biosafety Level-3* standards. Fouchier’s research facility has the requisite formal approvals for the virus research being conducted there, including a permit from the Dutch government (2007) and U.S. Center for Disease Control (2007) approvals of the security infrastructure of the laboratory. Further, the U.S. National Institutes of Health (NIAID) has funded Fouchier’s research with a seven-year commitment (sub-contract with Mt. Sinai School of Medicine awarded 2005, consequent to an NIH-NIAID-DMID-07-20 call for proposals). Fouchier’s facility includes use of *in vitro* class 3 biosafety cabinets and *in vivo* class 3 isolator units for the research on ferrets. (Personal notes, web-link access to AMS presentation; see reference [38]).

Kawaoka’s facility is designated BSL3-Ag (Biosafety Level 3-Agriculture), which, according to U.S. Department of Agriculture (USDA) standards, requires inventory control procedures, physical security systems, cybersecurity systems, personnel suitability, a biosecurity incident response plan, and ongoing federal oversight—standards deemed essential for zoonotic pathogens (causing disease in both animals and humans), avian influenza virus included as a pathogen of “high consequence” (HCP) [39]. BSL3-Ag requires “adding filtration of supply and exhaust air, sewage decontamination, exit personnel showers, and facility integrity testing” to reduce “the risk of environmental exposure to pathogens of consequence to agriculture.” According to the CDC designation, the BSL-3 level of containment is “applicable to clinical, diagnostic, teaching, research, or production facilities in which work is done with indigenous or exotic agents with a potential for respiratory transmission, and which may cause serious and potentially lethal infection” [37]. The principal investigators, seeking to provide assurance to the public, are aware of “a perceived fear that the ferret-transmissible H5HA viruses may escape from the laboratories;” but they note that “these experiments have been conducted with appropriate regulatory oversight in secure containment facilities by highly trained and responsible personnel to minimize any risk of accidental release” (Personal notes, web-link access to AMS presentation; see reference [38]). They concede further that, “Whether the ferret-adapted influenza viruses have the ability to transmit from human to human cannot be tested.”

Some critics of the H5N1 research have argued that any continuation of the research should occur, as a matter of prudence and in view of some regularity of “lab accidents”, only in a BSL-4 level facility [40]. Imperiale and Hanna reconfigure the issue by considering whether making the H5N1 virus transmissible among mammals changes “its biosafety profile” [41]. Appealing to the precautionary principle, they argue for moving such research to BSL4 containment. Others caution against such a move because “BSL4 facilities are few in number and already engaged in research with numerous other pathogens” [42]. It is argued further that such a move would “make society potentially more vulnerable, since critical experimental work will not get done…,” thereby affecting influenza preparedness negatively as well as reducing opportunities for pharmaceutical companies to continue with vaccine R&D. Adolfo García-Sastre, the principal investigator for the NIH/NIAID research award sub-contracted to both Kawaoka and Fouchier, accounts for the fact that the Kawaoka and Fouchier experimental results have now ruled out a hypothesis widely held by influenza virologists, viz., that “H5N1 viruses might be structurally unfit for mammalian transmission” [43]. Given the need for continued research on “molecular mechanisms responsible for host specificity” such research, he argues, can be safely performed as now with BSL3 facilities.

Vincent Racaniello, (Professor of Microbiology and Immunology, Columbia University College of Physicians and Surgeons), objected to the initial NSABB assessment, finding the argument faulty for its assumption that experimental results in ferrets predict what may occur in humans [44]. He reminds that the reduction of virus virulence in humans occurs through passage of viruses in a different host. For example, “The infectious, attenuated yellow-fever virus and the poliovirus vaccines have been produced in this way. So, the possibility exists that passage of the H5N1 virus in ferrets will attenuate its virulence in humans, a possibility not considered by the advisory board.” Moreover, Racaniello complained of scientific hubris in predictive claims of critics of this research. Such engineered viruses, he argues, are not subject to the strong selection pressures, thus a natural process cannot be expected to be reproducible in the laboratory. Indeed, Horimoto and Kawaoka have commented that these viruses lack efficient replication in humans, meaning then that direct transmission of such viruses to humans would be “a rare event,” although there are some isolated incidents that argue against this [8]. Further, the authors hypothesized that, “Most probably, additional mutations introduced through continued replication in humans or perhaps reassortment with a currently circulating human virus will be required to produce a highly virulent and contagious virus” [8].

Influenza is among the viral diseases listed by the CDC in relation to biosafety standards [37]. Influenza is associated with epidemic disease frequency and can have high mortality rates, depending on the strain, with pandemic presence due to “reassortment of human and avian influenza virus genes,” i.e., mutation. Avian influenza represents one antigenic subtype that occurs naturally in wild avian species and domestic fowl. It is known that, “The human influenza viruses responsible for the 1918, 1957, and 1968 pandemics contained gene segments closely related to those avian influenza viruses” [37]. In its containment recommendations, CDC identifies the “primary laboratory hazard” as “inhalation of virus from aerosols generated by infecting animals or by aspirating, dispensing, mixing, centrifuging or otherwise manipulating virus-infected samples” [37]. Further, “genetic manipulation has the potential for altering the host range, pathogenicity, and antigenic composition of influenza viruses.” Biosafety level 2 is recommended for “low pathogenicity avian influenza (LPAI) strains,” while “highly pathogenic avian influenza (HPAI)” research is expected to follow Biosafety Level 3 protocol [37]. The USDA stipulates BSL-4 as the highest category of biosecurity facility, “applicable for work with dangerous and exotic agents that pose a high individual risk of life-threatening disease, which may be transmitted via the aerosol route and for which there is no available vaccine or therapy.”

Thus, under regulations currently in place in the USA and the Netherlands where the H5N1 research is being done, both research teams are compliant with containment standards.

### Research justifications and objections

According to Anthony Fauci, NIH/NIAID influenza research funding increased substantially from US$196 million in FY2006 to US$261 million in FY2007, consequent to heightened concern for both *host adaptation* and *transmissibility* of pandemic influenza viruses and the H5N1 threat in particular. (Personal notes, web-link access to AMS presentation; see reference [45]). The Fouchier and Kawaoka research engage both of these NIH/NIAID public health concerns, consistent further with the NIAID Influenza Blue Ribbon Panel (2006) and the World Health Organization (WHO) Research Agenda for Influenza (2009). Thus, on research initiation it was understood, by all concerned with protocol review and approvals, that *the two research protocols were (a) properly situated within, and (b) technically undertaken consistent with, both a national (USA, Netherlands) and internationally recognized (WHO, CDC) influenza research framework.*

The WHO expressed concern about the two experiments, but expected such studies could proceed under appropriate conditions, on the grounds that “critical scientific knowledge needed to reduce the risks posed by the H5N1 virus continues to increase” [46]. As noted above, public health officials are particularly concerned about H5N1 adaptation through antigenic variation, hence the annual pressure for new vaccines. Notwithstanding, the WHO cautioned about this research being done only after accounting for public health risks/benefits. Also, this research should not in any way undermine the Pandemic Influenza Preparedness (PIP) Framework put in place in May 2011. In light of the pressures for redaction of the Kawaoka and Fouchier submitted manuscripts, on 17–18 February 2012, a WHO-convened panel of influenza experts recommended “full disclosure,” with some delay, with the additional recommendation to extend the temporary moratorium on research with new laboratory-modified H5N1 viruses [47].

Risk reduction in this case includes not only what precautions are taken in laboratory infrastructure during the research endeavor, but also risk reduction in terms of the larger concern for national and global response preparedness in the event of a pandemic. The U.S. NIAID, for example, comments on influenza with reference to the techniques of “plasmid-based reverse genetics,” developed by Kawaoka [48]. Researchers believe this technique of reverse genetics can provide a vaccine sooner than what other methods allow, including for H5N1 influenza, because of assembly of genes with coding for desired features. Kawaoka believes this technique provides a way to “determine the pathogenic potential of a virus or its ability to cross the species barrier,” since the methods “allow the generation of an influenza virus entirely from cloned cDNAs” [49]. Further, Kawaoka argues that reverse-genetics vaccines have already been prepared according to World Health Organization (WHO) recommendations; in fact, there is already ongoing clinical evaluation in several countries, in which case the methods applied to H5N1 are reasonable strategies in vaccine research [50,51].

Both Kawaoka and Fouchier have commented on the significance of their work. Kawaoka is concerned to have some public health means of responding to an H5N1 pandemic [52]. Although H5N1 has “limited viral replication” traits, Kawaoka’s own research makes it clear that, “the factors that determine the interspecies transmission and pathogenicity of influenza viruses are still poorly understood” [53]. Further, Kawaoka’s research team reports, “an anatomical difference in the distribution in the human airway of the different binding molecules preferred by the avian and human influenza viruses,” which findings “may provide a rational explanation for why H5N1 viruses at present rarely infect and spread between humans although they can replicate efficiently in the lungs” [54].

Despite the 60-day moratorium on current research, Kawaoka argued for transmission studies such as his “with urgency,” given that “not all H5 HA-possessing viruses are lethal” [55]. That is a fact in favor of continued research, especially since in ferrets, the “mutant H5 HA/2009 virus was no more pathogenic than the pandemic 2009 virus—it did not kill any of the infected animals. And, importantly, current vaccines and antiviral compounds are effective against it.” Kawaoka rejected the NSABB risk assessment claims, countering that “H5N1 viruses circulating in nature already pose a threat, because influenza viruses mutate constantly and can cause pandemics with great losses of life… Because H5N1 mutations that confer transmissibility in mammals may emerge in nature,” Kawaoka opines that “it would be irresponsible not to study the underlying mechanisms” [55]. Kawaoka presented an argument, which may be structured thus:

•There is an urgent need to expand development, production and distribution of vaccines against H5 viruses, and to stockpile antiviral compounds.

•Both studies [Fouchier’s and Kawaoka’s] identify specific mutations in HA that confer transmissibility in ferrets to H5 HA-possessing viruses.

•A subset of these mutations has been detected in H5N1 viruses circulating in certain countries.

•It is, therefore, imperative that these viruses are monitored closely so that eradication efforts and countermeasures (such as vaccine-strain selection) can be focused on them, should they acquire transmissibility.

•Consequently the benefits of these studies—the knowledge that H5 HA-possessing viruses pose a risk and the ability to monitor them and develop countermeasures—outweigh the risks.

Such is Kawaoka’s counterargument to the NSABB position. Fouchier similarly commented on his motivations, noting that, “highly respected virologists…thought until a few years ago that H5N1 could never become airborne between mammals.” Not convinced, Fouchier decided “to make a virus that is transmissible” [56]. Further, Fouchier argued for the ability to predict behavior of the avian influenza virus, in the same way scientists work to improve techniques predicting earthquakes and tsunamis [57]. Fouchier believes that the results of such research should be published in detail without redaction, especially for those scientists responsible for influenza surveillance in countries affected by H5N1 [58].

Fouchier is concerned with response preparedness: “If those mutations would be detected in the field, then those countries affected should act very aggressively to stamp out the outbreaks, to protect the world.” Thus, Fouchier hopes to prevent a pandemic, having a “last resource” in antiviral drugs and vaccines [58]. As for the dual use implications, Fouchier recognizes that bio-terror or bio-warfare can be a problem, but he also believes using the H5N1 mutant virus is much too difficult—“there are so many easy ways of doing it that nobody would take this H5N1 virus and do this very difficult thing to achieve it. There are terrorist opportunities that are much, much easier than to genetically modify H5N1 bird flu virus that are probably much more effective.” Fouchier also contrasts risk assessment as measured by biosecurity experts and public health officials: “The only people who want to hold back [on this research] are the biosecurity experts. They show zero tolerance to risk. The public health specialists do not have this zero tolerance…” For Fouchier, research on the biological properties of the virus is of utmost importance, even more so than the mutations^b^[33].

Given the institutional approvals of the initial research protocol, and given the current stage of research results, Fouchier argues that, “detailed knowledge” of research results is required “to ensure implementation of the most up-to-date molecular diagnostics and virus genome sequence interpretation” [59]. Further, Fouchier claims, “Companies and research organizations with research and development programs aiming at the development of diagnostic tests, vaccines, and antiviral drugs for H5N1 virus need to know if the effectiveness of such tools depends on the virus lineage or specific mutations.” Moreover, Fouchier adds, “research laboratories that study H5N1 virus host adaptation, H5N1 virus in mammalian model systems, or use the same virus lineage that was the subject of our studies have a need to know because they may unknowingly develop high-risk variants.” Fouchier makes it clear that this is not merely hypothetical, given the fact of laboratories working with H5N1 viruses that may require only “one to three mutations before the viruses used may become transmissible via aerosols” [59]. Given these “need to know” claims, Fouchier’s team is therefore unequivocal: “We do not agree with the NSABB recommendations.”

Others involved in research with pathogens have provided their own arguments in this debate. Peter Palese’s research in the 1990s led to the reconstruction of a “live virus” version of the 1918 flu virus, which also entailed NSABB review but moved forward with full publication [60]. Palese argued in present case against censorship, by comparison to the consequences of publication of his research in 2005, which in his case turned out not to have the consequence of a “nefarious scientist” recreating a Spanish flu bioweapon. Notwithstanding, the question has been raised whether such research should have been undertaken in the first place with the claim that in this case the risks outweigh the benefits [61-63].

Michael Osterholm, an epidemiologist who is a member of the NSABB, argued against disclosure because of the bioterrorist potential. The problem for him is not a single instance of bioweapon use but instead what he calls the “echo impact”—“where the illnesses may occur for weeks after the initial hit… [and] where the transmission can occur from [one] generation to another” [64]. Despite his epidemiological expertise, and in contrast to other public health opinions, Osterholm argued for zero tolerance in this case [65]. Osterholm provided his argument through a series of hypothetical propositions with his conclusion, which can be structured thus:

•If this virus does readily transmit between humans, just as it’s now doing between ferrets, which, to date have been the best animal model we have for predicting its performance or behavior in humans; and,

•If in fact, this virus is as lethal in humans as it is in ferrets;

•*Then:* this would be as serious an infectious disease encounter that the human population has ever known…

•So we cannot afford to be even a little bit wrong here. Further,

•If we make a mistake, the virus either gets out accidentally or it is, in fact, developed by someone who has a nefarious purpose in mind, obviously we haven’t done our job.

More recently, consequent to NSABB’s decision to publish the two manuscripts without redaction, Osterholm wrote with continued objection [66]. He voted against full disclosure of methods and results in the Fouchier paper, concerned as he is that the revised manuscripts “are immediately and directly enabling” of replication. Added to his concern is his report that Fouchier “has already identified an additional mutation (not included in his current manuscript) that results in ferret-to-ferret transmission (mammalian transmission) without the need for repeated passage of the virus in ferrets.” Further, given his lab’s research on H1N1, which, he notes, recurred in 1977 after a 20-year absence from global circulation, Osterholm opines, “we are convinced [H1N1] leaked out of a Russian lab that was working on a live-attenuated H1N1 virus vaccine” [66]. The implication is that there is the same probability of accidental release of H5N1-Mut through prospective replication of Fouchier’s research.

### Engaging the moral issue of research design

The foregoing represents the contour of the debate on various points of concern. Commenting on “oversight, biosafety, and biosecurity,” Fouchier’s research team continues to remind that its work has been done “completely openly” following “local, national, and international consultation.” This included consultations with biosafety officers, facility managers, and virologists operating class 3 and class 4 facilities [58]. In addition to US Department of Health and Human Services review and a National Institutes of Health funding award, Fouchier’s team reported it was granted “an explicit permit to work with aerosol-transmissible H5N1 virus” from the Dutch Ministry for Infrastructure and Environment in 2007, including as part of that permit regulatory review from the Commission on Genetic Modification (COGEM), the latter concluding that “the proposed work could be performed with negligible risk to humans and the environment under the conditions outlined in the application.” Fouchier’s facility is further inspected by the U.S. Centers for Disease Control, with the most recent inspection having occurred in February 2011, “at which time no shortcomings in biosafety and biosecurity measures were identified.”

Thus, Fouchier’s team points to the normative question at issue here, and calls for a framework of reasoning, thus:

•First, there is a scientific question at issue here that is posed by the scientific and biosecurity specialists involved in this dispute: *If by some means, accidental or nefarious, the H5N1 laboratory mutated virus (H5N1-Mut) is released into the natural environment, how will H5N1-Mut virus behave in the natural environment?* This question, ***q***, we shall refer to as our “puzzle predicament, ***p***.” This question is a matter of scientific *prediction*, itself dependent on an assortment of empirical variables.

•Second, one may set forth a guiding proposition: *If one has a puzzle-predicament****p****, given****q****, then one no longer has****p****when one has a correct answer,****A****, to****q****.* But, logically, “Any answer not known to be false will do, even if not known to be true” [67].

Thus, we have at least two answers to the scientific question to consider:

•**A1:** H5N1-Mut *will behave* in humans with the same lethal pathogenicity it manifests in ferrets maintained under laboratory conditions in the Fouchier lab.

•**A2:** H5N1-Mut *will not behave* in humans with the same lethal pathogenicity it manifests in ferrets maintained under laboratory conditions in the Fouchier lab.

As of current date, *each of the two above answers is not known to be false*, even as *neither is known to be true.* Thus, the truth-value of each proposition is not known.

Epistemologically, this latter claim is different from the claim that the truth-value of each proposition is *undecidable.* Accordingly, one may reasonably ask whether there is some means by which we have evidence one way or the other to decide the truth-value of either **A1** or **A2** and so have a correct answer to our puzzle-predicament, ***p***. Since we are engaged by empirical questions and expect empirically supported evidence, a correct answer will be only inductively probable and not certain. In short, given our present state of ignorance, the scientists involved in the H5N1-Mut debate would have to consider the question: “what methods for carrying out vicarious investigations are offered?” We must bear in mind that this latter question is reasonably posed only on the assumption that *the truth-value of***A1***or***A2***is decidable* through the presentation of some kind of empirical evidence. Our task here is, at the least, to eliminate (if not eradicate) our ignorance concerning the answer to our question, ***q.***

Sylvain Bromberger characterizes a “rational ignoramus” as someone who “must deliberately select from his ignorance at time *t* one question for elimination,” in which case it is presupposed this person “must be able to survey the membership of his ignorance” and, thereby, “establish his preferences” as to the questions to be eliminated. The scientists debating the prospective behavior of H5N1-Mut are, in effect, stipulating either **A1** or **A2** as the primary answers of interest, consistent with their given attitude towards risk assessment—which is either wholly *risk intolerant* (some biosafety experts such as Osterholm, Keim, and Henderson) or *risk tolerant to a degree* (some public health officials, principal investigators such as Fouchier and Kawaoka, and other scientists such as Palese and Racaniello). We can only *assume* (rather than *know*) these scientists are able to determine the truth-conditions for **A1** and **A2**, i.e., what else empirically must be true (or reasonably probably true) if **A1** is true or, alternatively, if **A2** is true.

For the time being, clearly, we need not concern ourselves with every possible answer to our question, ***q***. What matters to the scientific community and to the public interest at this time is an answer to ***q*** stated as either **A1** or **A2**. And, either answer will (or should) involve evidence “strong enough to warrant the belief that one knows the answer” to be **A1** or **A2**[67]. That is to say, for both **A1** and **A2** one must reasonably have a set of *justified true beliefs* to accept either **A1** or **A2** as a correct answer to ***q***. Justified true beliefs include having reasons for or against the continued pursuit of this research, even as such reasons should have been in place at the outset when this research was first authorized. Following an analytical scheme presented in the work of philosopher Derek Parfit, it may be said that these reasons can be *decisive*, even *strongly decisive*, in relation to the options of proceeding with or foregoing this kind of research [68].

Individuals, including scientists, can have both true beliefs and false beliefs, either or both of which (severally or jointly) may be articulated as reasons for or against various actions. Scientists may have justification or warrant for their true beliefs. They may even provide what is purported to be justification or warrant for what, unknown to them, are false beliefs. An influenza virologist, for example, may *believe* that (**A**) the H5N1-Mut virus, if released into the natural environment (whether through accident or nefarious means), will have the same pathogenicity manifest in the ferret model in the event of aerosol transmission of this virus among humans; and, accordingly, *believe further* that (**B**) the combination of aerosol transmission and high virulence of H5N1-Mut in humans will result in a pandemic of H5N1 influenza with a mortality rate far exceeding that of the 1918 influenza pandemic. Such are likely to be the beliefs of one such as Osterholm. But, one would have to ask what it means to have such beliefs, i.e., beliefs about *predictions* that are dependent on any number of hypotheses and empirical variables, in contrast to having a belief about a *fact* that is already inductively warranted and which has reliable empirical grounding. How does one *test* such a prediction if it is to be a guide to decision-making that has public health and science policy consequences?

A philosopher such as W. V. O. Quine would argue that, “Testing a given empirical prediction to the satisfaction of the scientific community requires only that there be a sufficient context of shared background assumptions to provide the rules for the empirical test” [68]. But, in the case of H5N1-Mut research we have no international consensus as to the background assumptions or rules for empirical testing. Regarding evidence of H5N1-Mut pathogenicity and efficiency of transmission, we have only *in vivo* data from the ferret models used by Fouchier and Kawaoka, combined with questionable serological surveillance data and debated human case-fatality data, to inform us about a plausible empirical test, on the basis of which inferences to prospectively probable human transmission may then be made. But, the level of ambiguity—and even disagreement—about probability are thereby wholly problematic, almost to the point of total ignorance.

If one’s epistemological situation is one of ignorance concerning a future event (i.e., one simply *does not know* either **A** or **B** above, although one *believes* by way of hypothesis that either **A** or **B**), then what a scientist should or should not do, as a matter of morally warranted research integrity, reasonably comes into *normative conflict* in relation to an epistemic state. That is, this normative conflict in this case is entirely likely to be articulated as *a conflict among the scientists having differing beliefs about the hypothesized future events****A****and****B****.* This is clearly evident now among influenza virologists and biosafety experts (the latter including infectious disease specialists), who differ in the strength of their convictions on the answer to a question involving a prediction about the pathogenicity and transmissibility of H5N1-Mut in humans. Thus, we are faced with a central epistemological question, the answer to which is a presupposition to the ongoing debate about the science involved: Does the proposition representing a prediction, in and of itself, have *truth-value*? That is, is it *decidable* whether this proposition represents a *true belief* rather than a *false belief*?

One may surely argue that **A** and **B** propositions stated above are both *possible* empirical outcomes, but that neither **A** nor **B** is a *necessary* empirical outcome. That is, **A** or **B** is each contingent on any number of variables that can make one or the other proposition more or less *probably true* or more or less *probably false* but *not necessarily/apodictically* true or false. Depending on one’s theory of truth, one may even go so far as to argue that **A** and **B** do not have *truth*-value at all when proposed, but that instead they have only a *probability*-value intermediate between truth (value T = 1) and falsehood (value F = 0), with (T = 1) and (F = 0) being inclusive as prospective empirical outcomes [69]. A value <1 then “signifies objective *chance*” such that, “If ‘1’ means ‘known’, [then] a value short of 1 signifies a degree of (subjective) *credence* or epistemic support.” But, even then, one would have to consider the presumed symmetry of intuitions operative here, according to which one individual reasonably prefers to think the predictive proposition to be *neither true nor false* while another’s intuition is that the predictive proposition is *either true or false*[70]. MacFarlane has argued that, “an adequate account of future contingents must respect both these intuitions;” and, further, “in order to make good sense of future contingents, we must allow the truth of utterances to be relativized to the context from which they are being assessed” [71]. Thus, it is coherent to say, “an utterance is true [false] with respect to a context of assessment *a* iff the sentence uttered is true [false] with respect to a context of utterance and *a* [context of assessment]” [71].

Consider, by way of illustration: If one says *predictively*, ‘tomorrow will be the end of the world’, and one *believes* ‘tomorrow will be the end of the world’ to be *true today* because one believes ‘*tomorrow will prove the proposition true*’, then one can say these are two beliefs which indeed one truly holds. But, these are not therefore also beliefs one *knows* (infallibly) to be true; nor are these beliefs which one can expect others to hold to be true merely because one holds these beliefs (assuming no fallacy of appeal to authority in this case). It can be a *fact* that one truly holds the two beliefs; but this fact *in and of itself* does not entail the *yet-to-be-demonstrated* claim that ‘tomorrow will be the end of the world’. By parity of reasoning, then, one would have to say the same in the case of predictive argument (beliefs) concerning the pathogenicity and transmissibility of H5N1-Mut once released into the natural environment. It is a *fact* that biosafety experts truly hold the *belief****(A)*** that H5N1-Mut, if accidentally or nefariously released into the environment, will have the same efficiency of transmission and the same pathogenicity in humans as that observed in ferrets. But, the fact of biosafety experts having this belief, in and of itself, does not entail the *yet-to-be-demonstrated fact* that, H5N1-Mut, if accidentally or nefariously released into the environment, will have the same efficiency of transmission and the same pathogenicity in humans as that observed in ferrets. And, of course, it is precisely this *prospective* fact that all do not want to see demonstrated, no matter the prediction.

Suppose we take both Osterholm (*et. al.*) and Fouchier (*et. al.*) as utilitarians faced with the decision whether (1) to publish with redaction (such as NSABB proposed) or (2) to publish with full disclosure (such as Fouchier preferred). The former finds full publication a severe disutility, due to the prospect of accidental release and/or nefarious use of H5N1-Mut consequent to replication of the research. The latter finds full publication a significant utility, due to the prospect of improvements in knowledge in avian influenza virology and epidemiological surveillance associated with mutation of H5N1*Wt*, etc., based on what one may learn from continued research with H5N1-Mut. The issue here is first of all *epistemic*, and then, secondly, ethical. That is, one may be concerned reasonably to evaluate *the rationality of the beliefs* held by Osterholm and Fouchier, associated as these beliefs are with the two outcomes being debated, whatever the reliability of the predictions and the epistemic value of those predictions. One may thereafter be concerned to evaluate *the ethical judgment* and proposed *morally warranted action* that are consequents of the beliefs held. If we are to consider the beliefs rational, we expect that each scientist has either an implicit or explicit commitment to a *probability measure* of outcome predicted, although it is unclear whether that measure is quantitative or qualitative. NSABB’s position fails to disclose both the basis and the analysis of risk/benefit and merely expects the public at large to accept this on authority, which is unacceptable in a matter such as this one affecting the public interest.

Each of the probability measures (whatever they may be) is related to a belief that Osterholm holds and a belief that Fouchier holds. We do not say these beliefs are irrational, given the analyses of utility and disutility each has identified, as partially surveyed in the representation of the (public) debate heretofore. But, surely, one may expect that one of these two positions is *dominant* by some measure of *maximal expected* utility. Fouchier surely holds that it is rational for him to conduct the research in a BSL-3 facility if he believes (as he does) that there is a probability of an accidental release of H5N1-Mut even as he allows for a probability of nefarious use consequent to a full disclosure of the research in an open access publication venue. But, in his case, Fouchier finds the maximal expected utility in continuing the research with open disclosure, rather than in continued research with redacted disclosure. Osterholm, in contrast, surely holds that it is rational for Fouchier and Kawaoka to conduct the research in a BSL-3 facility if he believes (as he does) that there is a probability of an accidental release of H5N1-Mut even as he allows for a probability of nefarious use consequent to full disclosure of the research in an open access publication venue. But, in his case, Osterholm finds the maximal expected utility in continuing the research but, if and only if there is redacted disclosure of the research methods. Osterholm is less concerned with the prospect of accidental release than he is with nefarious use, in which case his probability measure would have to weigh the former lower than the latter.

### Applying Parfit’s argument

Clearly, with the utilitarian/consequentialist approach to estimation of risk/benefit, we find ourselves in a continuing conundrum, without resolution of the debate in favor of either redacted disclosure such as NSABB recommended or open disclosure such as the influenza virologists preferred. Some other analytical approach is, therefore, in order. NSABB authorities would have contributed immensely to resolution of the debate more efficiently had they disclosed the basis of their risk/benefit analysis. All would likely benefit from an epistemic utility analysis that has quantitative methods in use adding to qualitative analysis. This is not the place for this exercise, especially in the absence of the data available to NSABB review. Instead, I turn to the philosopher Parfit to engage the epistemic question differently.

Following Parfit, we can say that acting on the basis of a true belief is rational; acting on the basis of a false belief is irrational. Such action presupposes one having *practical* reasons. One may, indeed, say that sometimes individuals (including otherwise competent scientists whose research integrity is by no means to be impugned) act on the basis of false beliefs. But, logically (according to Parfit), “if we say that false beliefs can give people reasons, we would need to add that these reasons do not have any *normative force*, in the sense that they do not count in favour of any act. And, we would have to ignore such reasons when we are trying to decide what someone has most reason to do” [68]. But, Parfit prefers instead that we distinguish *apparent* reasons from *real* reasons, in which case we would say that “all reasons have normative force;” and, further, “When we give people advice, we can ignore the merely apparent reasons that are provided by these people’s false beliefs.”

Following Parfit, then, in the case of virologists’ prediction of H5N1-Mut behavior in the natural environment, one would thus have to distinguish apparent reasons and real reasons, ignoring the apparent reasons when having to decide, e.g., whether to recommend continuation of H5N1-Mut research. This is an important point, because, as Barrett and Stanford say, “our predictive language has continuously respected the fundamental idea that a prediction is a claim about unknown matters of fact whose truth or falsity has not already been independently ascertained by some more direct method than that used to make the prediction itself” [69]. Thus, prediction is “inherently risky” and subject to refutation, given the defeasible character of scientific reasoning—i.e., the introduction of new evidence subjects the prediction to defeat and thus to elimination of the warrant it supposedly had.

Such risk must be evaluated in terms of its epistemological character. Parfit argues that individuals “might be practically rational but epistemologically irrational, or practically irrational but epistemologically rational” [68]. The scientific question at issue for both the virologists and the biosafety experts is not merely one of epistemic rationality (which, Parfit says, has “the aim of reaching true beliefs”). Instead, it is one of practical rationality, i.e., the aim is that of doing something, something that is hopefully correct. The influenza virologists are hard at work on H5N1Wt and, now, H5N1-Mut for both *epistemic* reasons (e.g., to know the molecular and biological variables associated with the virus) even as they have *practical* reasons for pursuing this research (e.g., working out pathogenesis and host response for the practical public health goals of improved influenza surveillance and prospectively successful R&D in vaccine and antiviral therapy).

If we take the NSABB recommendation to redact the journal articles to avoid disclosure of research methods (based on NSABB’s concern about prospective nefarious dual-use consequences of the research at issue), then we could say (as Parfit does): “…acts are best called irrational only when, in…acting in some way, we are failing to respond to clear and strongly decisive *practical* reasons or apparent reasons not to…act in this way” [68]. Thus, we have here a main point of argument, to be structured thus:

•*Fouchier’s or Kawaoka’s continuation of their research or full disclosure of their research methods and results would properly be called irrational only when, in doing so, Fouchier and Kawaoka fail to respond to clear and strongly decisive practical reasons not to pursue that research or not to publish fully.*

•Given the survey of dispute reviewed in the first two sections of this paper, one may reasonably assert that *it is not the case that either scientist, Fouchier or Kawaoka, has clear and strongly decisive practical reasons presented to them by NSABB or any other public health or biosecurity authority for not pursuing their research.*

•Hence, neither Fouchier nor Kawaoka can be characterized as irrational in deciding to continue their research endeavor or in deciding to publish their research in full detail.

On the other hand, if we take Paul Keim’s assertion that the *probability* of nefarious use of the H5N1-Mut virus is *low*, we might still ask whether this low probability (assuming it to be correct) is to be reasonably ignored. In “consequentialist terms,” Parfit has remarked, “When I cannot predict the effects of my act, [consequentialism] tells me to do whatever would produce the greatest *expected* benefit. The expected benefit of my act is the possible benefit multiplied by the chance that my act will produce it” [72]. The “possible benefit” in this case is (*Benefit 1*) no nefarious release of H5N1-Mut into the natural environment and, consequently, (*Benefit 2*) no evolutionary reassortment of the virus to the detriment of global public health through an H5N1-Mut pandemic. Multiply this possible benefit by the probability that (a) full disclosure of the Fouchier and Kawaoka research methods is prohibited, and (b) replication and validation of these research results are thereby also prevented. One must then ask: Will the consequence(s) of these two proposed actions represent the achievement of the expected benefit? That is, will the consequences be substantively *positive*, rather than negative, in the calculation (accounting here for the virologists’ contribution as defined by the practical reasons motivating their research)?

To answer the question, one may draw a comparison here. Parfit [72] illustrates the point with this example: “Suppose that nuclear engineers did ignore all chances at or below the threshold of one-in-a-million. It might then be the case that, for each of the many components in a nuclear reactor, there is a one-in-a-million chance that, in any day, this component would fail in a way that would cause a catastrophe. It would be clearly wrong for those who design reactors to ignore such tiny chances. If there are many reactors, each with many such components, it would not take many days before the one-in-a-million risk had been run a million times. There would fairly soon be a catastrophe.” Thus, Parfit asserts: “When the stakes are very high, no chance, however small, should be ignored… We can usually ignore a very small chance. But we should not do so when we may affect a very large number of people, or when the chance will be taken in a very large number of times. These large numbers roughly cancel out the smallness of the chance.”

The question of chance here, in the case of avian influenza virology, requires some reference to the evolutionary biology associated with virus mutation. One may consider here that evolutionary biology treats the process of mutation as “a random variable…either because it is genuinely indeterminate or because we don’t know yet enough about the process or relevant conditions in particular organisms to predict what, when and how particular mutations will occur with any precision” [73]. In present case of H5N1*Wt* naturally mutating to the natural genetic equivalent of the laboratory-created H5N1-*Mut*, we may or may not now know enough (consequent to the Fouchier and Kawaoka experiments) about either the *process* or the *conditions* in H5N1*Wt* to predict *when* and *how* this strain will reassort naturally for H5N1-Mut to start showing up internationally in sero-epidemiological surveillance. If the mutation were to occur naturally and sero-epidemiological evidence began to show this, there is little doubt that a large number of people would be affected, hence the problem of a high-mortality pandemic in the absence of cross-protective immunity from other influenza infections.

It is this “chance” that the NSABB panel has argued we cannot take. However, as long as the research with H5N1-Mut is restricted to BSL-3 labs, the chance of accidental release will not be taken a very large number of times (in terms of the number of BSL-3 facilities worldwide having the capacity or agenda for such research), in which case this low probability provides some guidance for at least a regulatory decision that, at minimum, such research should have adequate oversight to assure its restriction to BSL-3 facilities. Such restriction clearly does not dismiss the probability of accidental release, although in the case of these facilities the probability is quite low so as to overcome an argument against continued research. The only “chance” concern, then, is the probability of natural evolution of H5N1*Wt* to an aerosol transmissible strain. But, if the concern is less with accidental release or nefarious use of H5N1-Mut and more with this probable evolution of H5N1-*Wt* to an aerosol transmissible strain, then we have additional reason to permit ongoing H5N1-Mut research for all the reasons already specified by Fouchier and Kawaoka. Ignorance is far more risky than study of these variables and unknowns under the regulatory conditions now in place for BSL-3 facilities.

Those involved in future studies have held that “the nature of predictive arguments is evidential” rather than demonstrative [74]. Further, the logic of prediction is different from that of explanation. Empirical adequacy, for example, in Bas Van Fraasen’s sense, requires that future observable consequences be true even as past and present observable consequences are true [75]. Yet, this is surely problematic for most fields of scientific research, including in present case predictive claims in microbiology and virology concerning the pathogenicity and transmissibility of a given pathogen, such as predictive claims now at issue in the H5N1-Mut debate. In the present debate there is a need for caution and restraint in merely deferring to the professional authority of either the influenza scientists or the biosecurity experts, if only because “the increased demand for policy-relevant scientific prediction has not been accompanied by adequate understanding of the appropriate use of prediction in policy making” [76].

## Conclusion

NSABB reported that it had calculated risks and benefits associated with full disclosure of the H5N1-Mut research. In this sense, NSABB authorities have been utilitarian/consequentialist in their analyses. However, it is problematic, to say the least, that NSABB has done so without disclosing those details to the public at large, which diminishes the authority of its assessment in the absence of appropriate public validation. Such calculations are more likely to be qualitative rather than quantitative (even allowing for statistical sophistication provided by epidemiological methods). This is why one has to be concerned about both empirical adequacy and reliability of such risk/benefit claims, such as discussed in the preceding section. In H5N1 laboratory research and in associated influenza field surveillance, we do not have invariant phenomena that are being explained, and which stand in a relation of consistent observations to consistently validated predictions. Instead, prediction here must account for the *variant* behavior of pathogens according to any number of variables not readily controlled, e.g., the naturally occurring mutation of a virus (e.g., H5N1-Wt naturally evolving to the lab-equivalent H5N1-Mut) in a particular reservoir (avian species), this virus then adapting to (or expanding) its host range (e.g., from avian species to mammals) in a particular geographic setting (Indonesia, Bangladesh, China, etc.).

Given the qualitative/quantitative distinction in relation to calculation of risk/benefit, the NSABB position about the Fouchier and Kawaoka experiments is a case in point of what Sarewitz and Pielke, Jr., have recognized to be “the political role” associated with scientific prediction. This role is characteristically “seductive,”: “If predictive science can improve policy outcomes by guiding policy choices,” Sarewitz and Pielke, Jr. opine, “then it can as well reduce the need for divisive debate and contentious decision-making based on subjective values and interests. Prediction, that is, can become a substitute for political and moral discourse” [76]. What the NSABB debate in this case makes clear is that, reduction of debate, however, is an unacceptable outcome precisely because it *a priori* privileges the authority of “the scientific estate” to the detriment of a strong relation of ethics and science policy that should not be eliminated, precisely because of the need for reasonable moral governance amidst the growing complexity of scientific research, a complexity that includes ambiguous and contested epistemic states of the scientists involved. It behooves the public at large to maintain vigilance of the fact that there are limits to expert knowledge, including that of epidemiologists, influenza virologists, and biosecurity analysts. One should not merely defer *a priori* to scientific authority [77]. The public interest requires informed contribution from those who advocate for that interest as one of concern for the public common good and the long-term public benefit. Here, with the assessment carried out in the discussion of this analysis, the recommendation is clear: Given an *epistemological* assessment in relation to a *moral* assessment where facts, predictions, and moral standards are taken into account, neither Fouchier nor Kawaoka can be characterized as irrational in deciding to continue their research endeavor or in deciding to publish their research in full detail. In this case, there is moral warrant in place reasonably to permit genetic alteration of the H5N1 strain of avian influenza for the purposes presented in the research protocol.

Of course, the contrary argument can be made, insofar as a position such as that of Osterholm is advanced as likewise not irrational (according to the structure of reasoning provided by Parfit), given our epistemological position in relation to known and unknown facts and variables. However, the standard of open research must be faced with considerable empirical and/or moral argument against it, to counter the validity of the standard of open disclosure, even in the case of H5N1-Mut research. Neither empirical nor moral argument (as surveyed hitherto) is sufficiently countervailing to defeat the validity of this standard for this type of research. Moreover, in the absence of reliable, more or less universalist, forms of argument, and given the possibilities in research outcome that are at issue in this research, *permissibility* rather than prohibition seems the weightier option (as would be clear even with a probabilist type of reasoning such as is delivered in casuistry’s attention to licit, rather than illicit, action, and such as may be issued in a rigorous epistemic utility analysis that NSABB authorities could have produced in defense of their recommendations). Hence, the final decision to permit full disclosure and publication of the Fouchier and Kawaoka papers is consistent with the analysis presented here.

## Endnotes

^a^“Fouchier and colleagues used a combination approach, engineering the virus and then stepping back to let nature take its course. They introduced key mutations into H5N1’s genetic code and then infected the ferrets…Typically…this is done by placing the virus in ferret noses, waiting a few days, swabbing out some mucous, infecting another ferret with it, and repeating the process over and over. Throughout the process, infected and uininfected ferrets would be placed in adjacent cages to see whether the virus could pass from one animal to the other without them touching… [Just] five tweaks in two genes, followed by just 10 passages of the virus between ferrets, created a pathogen that could travel through the air from animal to animal. The virus remained lethal.” (Brown, *Los Angeles Times*, 26 December 2011).

^b^Fouchier speaks of the virus being attracted to the upper respiratory tract rather than in the lung; whether the virus binds to certain mammalian receptors; whether it has to reproduce in large amounts to increase chance of transmission; whether it has to be stable in small droplets; etc., all of which variables vary yet further with “more possible mutations.” For technical comments, see E.M. Sorrell [62]. Moreover, Fouchier has worked with only one genetic lineage of H5N1, in which case: “The question is whether all lineages can become aerosol-transmissible. If they can’t, if it’s just this lineage, perhaps you can focus on the region where it came from and try to stop H5N1 outbreaks there to prevent a pandemic. If it can happen everywhere, you’ve got to work everywhere.”

## Competing interests

No financial or non-financial competing interests.

## References

[B1] Editorial. An engineered doomsday 2012 The New York Times http://www.nytimes.com/2012/01/08/opinion/sunday/an-engineered-doomsday.html?_r=0. Accessed 25 February 2012

[B2] Osterholm Michael T Henderson Donald A Life sciences at a crossroads: respiratory transmissible H5N1 Science 2012 335 10.1126/science.1218612 22267584

[B3] Berns Kenneth I Adaptations of avian flu virus are a cause for concern Nature 2012 http://www.nature.com/nature/journal/vaop/ncurrent/full/482153a.html accessed 02 February 2012 10.1038/482153a 22294204

[B4] Fidler David P Risky research and human health: the influenza H5N1 research controversy and international Law Insights (American Society of International Law) 2012 16 2

[B5] Fouchier R ASM biodefense and emerging diseases research meeting, panel discussion 2012 https://www.asm.org/index.php/membership1/71-membership/archives/834-biodefense-meetings-historical-list

[B6] Cohen J Malakoff D NSABB members react to request for second look at H5N1 Flu studies ScienceInsider 2012

[B7] Kerkhove V Maria D Mumford E Mounts AW Joseph Bresee AW Sowath L Bridges CB Otte J Highly pathogenic avian influenza (H5N1): pathways of exposure at the animal-human interface, a systematic review PLoS One 2011 6 1 e14582 10.1371/journal.pone.0014582 21283678 PMC3025925

[B8] Horimoto T Kawaoka Y Pandemic threat posed by avian influenza A viruses Clin Microbiol Rev 2001 14 1 129 149 10.1128/CMR.14.1.129-149.2001 11148006 PMC88966

[B9] Eckroade RJ Bachin LAS Proceedings of the second international symposium on avian influenza Proceedings of the second international symposium on avian influenza 1987 22 32

[B10] Commission of the European Communities Impact assessment avian influenza. COM 171

[B11] Macleod A Economic and social impacts of avian influenza 2005 Food and Agricultural Organization http://www.fao.org/docs/eims/upload//211939/Economic-and-social-impacts-of-avian-influenza-Geneva.pdf

[B12] Perez Daniel R H5N1 Debates: hung up on the wrong questions Science 2012 Policy Forum. 335 10.1126/science.1219066 PMC5003609 22267585

[B13] Sims LD Avian influenza in Hong Kong 1997–2002 Avian Dis 2003 47 3 Suppl 832 838 14575073 10.1637/0005-2086-47.s3.832

[B14] Erasmus Medical Center Avian influenza could evolve into dangerous human virus 2011 http://www.erasmusmc.nl/corp_home/corp_news-center/2011/2011-11. Accessed 08 January 2012

[B15] Fouchier Ron A Sander H Albert DME O Restricted data on influenza H5N1 virus transmission. Science express policy forum 2012 http://www.sciencexpress.org. Accessed 07 February 2012

[B16] Roos R Live debate airs major divisions in H5N1 research battle Center for infectious disease research and policy 2012 http://www.cidrap.umn.edu. Accessed 07 February 2012

[B17] Wang Tala T Parides Michael K Palese P Seroevidence for H5N1 influenza infections in humans: meta-analysis Science 2012 http://www.sciencemag.org/content/early/2012/02/22/science.1218888. Accessed 25 February 2012 10.1126/science.1218888 PMC4160829 22362880

[B18] Fischman J Overstated risks of censored bird-flu papers—and overstated benefits 2012 The Chronicle of Higher Education

[B19] Osterholm Michael T Kelley Nicholas S Mammalian-transmissible H5N1 influenza: facts and perspective mBio 2012 3 2 published online 24 February 2012; Accessed 25 February 2012 10.1128/mBio.00045-12 PMC3302573 22366820

[B20] Roos R NSABB: studies show how H5N1 can jump natural barrier 2012 Center for Infectious Disease Research and Policy http://www.cidrap.umn.edu. Accessed 07 February 2012

[B21] Keim Paul S The NSABB recommendations: rationale, impact, and implications mBio 2012 3 1 http://mbio.asm.org. Accessed 11 February 2012 10.3391/mbi.2012.3.1.01 PMC3268663 22294677

[B22] Casadevall A Shenk T The H5N1 manuscript redaction controversy mBio 2012 3 1 http://mbio.asm.org. Accessed 11 February 2012 10.3391/mbi.2012.3.1.01 PMC3268664 22294678

[B23] Racaniello Vincent R Should science be in the public domain mBio 2012 3 1 http://mbio.asm.org. Accessed 11 February 2012 10.3391/mbi.2012.3.1.01 PMC3268661 22294675

[B24] Taubenberger Jeffery K Characterization of the 1918 influenza virus polymerase genes Nature 2005 437 6 889 892 16208372 10.1038/nature04230

[B25] Alberts B Statements regarding publication of HN1 avian influenza research Science 2011 http://www.aaas.org/news/releases/2011/1220herfst.shtml

[B26] Fouchier Ron A Garcia-Sastre A Kawaoka Y Correspondence: pause on avian flu transmission studies Nature 2012 481 20 443 10.1038/481443a 22266939

[B27] Fouchier Ron A Pause on avian flu transmission research Sci Expr 2012 http://www.scienceexpress.org 10.1126/science.335.6067.400 PMC3812248 22282787

[B28] Banerjee N NSC wants rules on research that could lead to biological weapons 2012 http://ecollegetimes.com/student-life/nsc-wants-rules-on-research. Accessed on 21 February 2012

[B29] Office of Biotechnology Activities, National Institutes of Health United States government policy for oversight of life sciences dual use research of concern http://oba.od.nih.gov/oba/biosecurity/PDF/United_States_Government_Policy_for_Oversight_of_DURC_FINAL_version_032812.pdf. Accessed 01 April 2012

[B30] Office of Science Policy Office of Biotechnology activities 2012 National Institutes of Health http://oba.od.nih.gov/biosecurity/about_nsabb.html. Accessed 04 January 2012

[B31] NSABB Meeting of the National Science Advisory Board for Biosecurity to review revised manuscripts on transmissibility of a/H5N1 influenza virus 2012

[B32] Imai M Experimental adaptation of an influenza H5HA confers respiratory droplet transmission to a reassortment H5HA/H1N1 virus in ferrets Nature 2012 published online 02 May 2012. http://www.nature.com/nature/journal/vaop/ncurrent/full/nature10831.html 10.1038/nature10831 PMC3388103 22722205

[B33] Herfst S Airborne transmission of influenza a/H5N1 virus between ferrets Science 2012 1534 1541 http://www.sciencemag.org/content/336/6088/1534.abstract 22723413 10.1126/science.1213362 PMC4810786

[B34] Enserink M Flu researcher Ron Fouchier: ‘It’s a pity that it has to come to this Science Insider 2012 http://news.sciencemag.org; Accessed 07 February 2012

[B35] Inglesby Thomas V Cicero A Henderson DA The risk of engineering a highly transmissible H5N1 virus Editorial 2012 Center for Biosecurity, University of Pittsburgh Medical Center http://www.upmc-biosecurity.org/website/resources/publications/2011/2011-12-15-editorial-engineering-H5N1; accessed 25 February 2012 10.1089/bsp.2011.1214 22176667

[B36] Ehni H-J Dual use and the ethical responsibility of scientists Arch Immunol Ther Exp (Warsz) 2008 56 147 152 10.1007/s00005-008-0020-7 18512027

[B37] Center for Disease Control Biosafety in microbiological and biomedical laboratories 2012 5 http://www.cdc.gov/biosafety/publications/bmbl5/index.htm. Accessed 07 January 2012

[B38] Fouchier R ASM biodefense and emerging diseases research meeting Panel Discussion 2012 https://www.asm.org/index.php/membership1/71-membership/archives/834-biodefense-meetings-historical-list

[B39] U.S. Department of Agriculture 2002 USDA security policies and procedures for biosafety level-3 facilities, departmental manual 2002 Washington D.C: Agricultural Research Service http://www.ocio.usda.gov/directives/doc/DM9610-001.htm; accessed 09 January 2012

[B40] Murillo Lisa N Ferret-transmissible influenza a(H5N1) virus: Let us err on the side of caution mBio 2012 3 2 10.1128/mBio.00037-12 PMC3302569 22396481

[B41] Imperiale Michael J Hanna III Michael G Biosafety considerations of mammalian-transmissible H5N1 influenza mBio 2012 3 2 10.1128/mBio.00043-12 PMC3302571 22396482

[B42] Casadevall A Shenk T Mammalian-transmissible H5N1 virus: containment level and case fatality ratio mBio 2012 3 2 1 2 10.1128/mBio.00054-12 PMC3302574 22396484

[B43] García-Sastre A Working safely with H5N1 viruses mBio 2012 3 2 1 2 10.1128/mBio.00049-12 PMC3302572 22396483

[B44] Racaniello V The real danger is research: an uneasy nexus between science and security 2012 The Chronicle of Higher Education, Commentary http://chronicle.com/article/The-Real-Danger-Is-to-Research/130345

[B45] Fauci A ASM biodefense and emerging diseases research meeting panel discussion 2012 https://www.asm.org/index.php/membership1/71-membership/archives/834-biodefense-meetings-historical-list

[B46] World Health Organization WHO concerned that new H5N1 influenza research could undermine the pandemic influenza preparedness framework 2011 Media Centre Statement http://www.who.int/mediacentre/news/statements/2011/pip_framework. Accessed 31 December 2011

[B47] World Health Organization Media centre news release. Public health, influenza experts agree H5N1 research critical, but extend delay 2012 http://www.who.int/mediacentre/news/releases/2012/h5n1_research_20120217/en/index.html#. Accessed 18 February 2012

[B48] National Institute of Allergy and Infectious Diseases (Influenza): reverse genetics: building Flu vaccines piece by piece 2012 http://www.niaid.nih.gov/topics/Flu/Research/vaccineResearch; Accessed 09 January 2012

[B49] Neumann G Hatta M Kawaoka Y Reverse genetics for the control of avian influenza Avian Dis 2003 47 3 Suppl 882 887 14575081 10.1637/0005-2086-47.s3.882

[B50] Horimoto T Kawaoka Y Strategies for developing vaccines against H5N1 influenza a viruses Trends Mol Med 2006 12 11 506 14 10.1016/j.molmed.2006.09.003 17011235

[B51] Neumann G Fujii K Kino Y Kawaoka Y An improved reverse genetics system for influenza a virus generation and its implications for vaccine production Proc Natl Acad Sci U S A 2005 102 46 16825 9 10.1073/pnas.0505587102 16267134 PMC1283806

[B52] Penn M Flight lessons 2006 Wisconsin 24 http://www.uwalumni.com/home/onwisconsin/archives/winter06/winter06.aspx

[B53] Neumann G Kawaoka Y Host range restriction and pathogenicity in the context of influenza pandemic Emerg Infect Dis 2006 12 6 881 886 10.3201/eid1206.051336 16707041 PMC3373033

[B54] Shinya K Ebina M Yamada S Ono M Kasai N Kawaoka Y Brief communications: avian flu: influenza virus receptors in the human airway Nature 2006 440 435 436 10.1038/440435a 16554799

[B55] Kawaoka Y H5N1: flu transmission work is urgent Nature 2012 482 155 http://www.nature.com/nature/journal/v482/n7384/full/nature10884.html. Accessed 11 February 2012 22278057 10.1038/nature10884

[B56] Grady D McNeil Donald G Debate persists on deadly flu made airborne 2011 The New York Times/International Herald Tribune http://www.nytimes.com/2011/12/27/science/debate-persists-on-the-deadly-flu-made-airbone.html?ref=health

[B57] Brown E Studies of deadly H5N1 bird flu mutations test scientific ethics Los Angeles Times http://articles.latimes.com/2011/dec/26/science/la-sci-bird-flu-20111227. Accessed 31 December 2011

[B58] Carvajal D Security in H5N1 bird flu study was paramount, scientist says 2011 The New York Times International Herald Tribune http://www.nytimes.com/2011/12/22/health/security-in-h5n1-bird-flu-study-was-paramount-scientist-says.html?pagewanted=all

[B59] Fouchier Ron A Sander H Albert DME O Restricted data on influenza H5N1 virus transmission restricted data on influenza H5N1 virus transmission Sci Expr Policy Forum 2012 http://www.sciencexpress.org. Accessed 07 February 2012

[B60] Palese P Don’t censor life-saving science Nature 2012 481 115 10.1038/481115a 22237069

[B61] Taubenherger Jeffery K Characterization of the 1918 influenza virus polymerase genes Nature 2005 437 889 893 10.1038/nature04230 16208372

[B62] Sorrell EM Predicting ‘airborne’ influenza viruses: (trans-)mission impossible? Curr OpinVirol 2011 1 635 642 10.1016/j.coviro.2011.07.003 PMC3311991 22440921

[B63] Van Aken J Is it wise to resurrect a deadly virus? Heredity 2007 98 1 2 10.1038/sj.hdy.6800911 17035950

[B64] Public Broadcasting System/WGBH Frontline: plague war: interview with Michael Osterholmhttp://www/pbs.org/wgbh/pages/frontline/shows/plague/interviews/osterholm; Accessed 07 February 2012

[B65] Living on earth. Transcript interview with Michael Osterholm 2012 http://www.loe.org/shows. Accessed 07 February 2012

[B66] Osterholm M Letter to Dr. Amy Patterson 2012 Associate Director for Science Policy, NIH http://wwww.scienceinsider/NSABB%20letter%20final2041212_3.pdf. (no longer accessible online)

[B67] Bromberger S Science and the forms of ignorance On what we know we Don’t know: explanation, theory, linguistics, and how questions shape them 1992 Chicago: The University of Chicago Press 117 122 Online version; http://cslipublications.stanford.edu/bromberger-corpus/On-What-We-Know-We-Don’t-Know.pdf

[B68] Parfit D On what matters volume 1 2011 1 Oxford: Oxford University Press

[B69] Barrett J Kyle SP Prediction The philosophy of science: An encyclopedia 2012 New York: Routledge

[B70] Kaufmann S Hastings R, Jackson B, Zvolenszky Z Probabilities of conditionals 2001 Ithaca: Cornell University: SALT XI 248 267

[B71] MacFarlane J Future contingents and relative truth The Philos Q 53 2003 212 321 336

[B72] Parfit D Reasons and persons 1984 Oxford: Clarendon

[B73] Pfeifer J Sarkar S The philosophy of science: An encyclopediahttp://philsci-archive.pitt.edu/1798/1/bio_info.pdf

[B74] Algica Paul D Prediction, explanation and the epistemology of future studies Futures 2003 35 10 1027 1040 10.1016/S0016-3287(03)00067-3

[B75] Fraasen Bas V The scientific image 1980 Oxford: Oxford University Press

[B76] Sarewitz D Pielke R Prediction in science and policy Technol Soc 1999 21 121 133 10.1016/S0160-791X(99)00002-0

[B77] Kleinman Daniel L Science and technology in society: from biotechnology to the internet 2005 Malden MA: Blackwell Publishing

